# A low-tech, low-cost method to capture point-source ammonia emissions and their potential use as a nitrogen fertiliser

**DOI:** 10.1371/journal.pone.0296679

**Published:** 2024-01-31

**Authors:** Nicholas Cowan, Daniel Ashwood, Julia Drewer, Galina Toteva, Mathew R. Heal

**Affiliations:** 1 UK Centre for Ecology and Hydrology, Bush Estate, Midlothian, United Kingdom; 2 School of Chemistry, The University of Edinburgh, Edinburgh, United Kingdom; 3 School of Geosciences, The University of Edinburgh, Edinburgh, United Kingdom; IISS: Indian Institute of Soil Science, INDIA

## Abstract

Rising global energy prices have led to increased costs of nitrogen (N) fertilisers for farmers, but N pollution (losses) from agricultural activities can account for over 50% of the nitrogen applied. This study assesses the feasibility of a low-cost and low-tech method of NH_3_ emission capture from an agricultural point source (chicken manure) using a water column bubbling technique, and its application as a fertiliser to several plant types. Solutions of i) nitric acid (HNO_3_), ii) calcium nitrate (Ca(NO_3_)_2_), iii) a mixture of Ca(NO_3_)_2_ and HNO_3_ and iv) deionised H_2_O were used to scrub NH_3_ from air pumped from a storage container containing chicken manure. We conclude that NH_3_ can be captured from manure using low-tech methods, and that solutions of common fertiliser compounds such as ammonium nitrate and calcium ammonium nitrate can be replicated by binding captured NH_3_ to solutions of nitrate. Our results suggest that dissolved calcium nitrate is just as effective at scrubbing NH_3_ from the atmosphere as nitric acid at low concentrations, but could do so at a near neutral pH. For use on common silage grass for livestock feed, all of the captured ammonium solutions significantly increased yields, including the ammonium only solution. However, the aquatic plants (*Taxiphyllum Barbieri* and *Salvinia auriculata)* did not respond favourably to a high ratio of NH_4_^+^ in solution, and in the case of *Salvinia auriculata*, the plant was significantly damaged by the ammonium only solution. In conclusion, we highlight that the capture and utilisation of NH_3_ emissions from point sources is possible using very basic apparatus and that if used correctly, this captured nitrogen can be stored and applied to crops in a variety of forms which could reduce reliance and cost of mineral fertiliser use.

## Introduction

Emissions of ammonia (NH_3_) gas have negative impacts on air quality and human health, primarily through the formation of fine-particulate matter (PM_2.5_) which causes cardiovascular and pulmonary damage [1, 2]. Deposition of NH_3_ to the wider environment can also result in higher nitrogen (N) pollution, which harms biodiversity in both terrestrial and aquatic environments and leads to eutrophication [3, 4]. While ammoniacal N has been used to some degree for centuries [5], the invention of the Haber-Bosch process (industrial synthesis of NH_3_ from atmospheric N) and the subsequent widescale application of mineral N fertilisers increased global emissions and exposure to NH_3_ exponentially [6].

Anthropogenic emissions of NH_3_ are largely influenced by agricultural activities, particularly the application of N fertilisers (e.g. urea) and the management of livestock waste (urine and manure), which together account for over half of all global NH_3_ emissions [7]. In the UK, agriculture accounts for approximately 87% emissions of NH_3_ (225.9 kt NH_3_ of a total of 259.2 kt NH_3_ [8, 9]). While application of N (mineral and animal waste) to agricultural fields results in large but relatively diffuse emissions (field scale or greater), large point sources, such as livestock barns, feedlots and manure heaps also contribute significantly to NH_3_ emissions [10]. Here, where ammonium (ammonia dissolved in water, NH_4_^+^) concentrations are highly concentrated in manure and urine and temperatures are typically higher, increased volatilisation of NH_3_ from these sources can significantly reduce the N content of manures before it is typically applied to fields as fertiliser (ranges from 3–60% [11, 12]).

Manure accounts for approximately half of all N applied to fields in the UK, accounting for 1021 kt N every year [13]. Based on current prices of mineral N fertiliser (AHDB, May 2023), one kg of N costs approximately £1.48 (down from highs of £2.22 in March 2022); therefore, it can be estimated that manure N content in the UK currently accounts for £1.51 billion of N fertiliser each year. The cost of NH_3_ emissions from animal manures is two-fold. Primarily, it is a loss of N to the farmer, as volatilised NH_3_ is no longer available as N fertiliser needed to sustain crop growth. Based on previous statements, an estimated £275 million worth of N is lost from farms annually in the UK in the form of volatilised NH_3_ (225.9 kt N yr^-1^). Secondly, there is a societal cost of the damage done by NH_3_ pollution. While it is certain that reducing pollution in the form of NH_3_ (and other reactive N compounds) would benefit society and ecosystems by reducing impact on human health and biodiversity, putting an exact price on this is difficult due to the complex and cascading nature of N in the environment [14, 15]. It has previously been estimated that the total cost of NH_3_ emissions in the EU (Including UK in 2008) were in the region of 10 to 120 billion euros annually, emphasising both the large magnitude of these costs, and the high associated uncertainty [16].

Methods to reduce NH_3_ emissions from animal waste have been developed, and there has been pressure on the agricultural industry to reduce and recycle emissions for some time [17]. Acidification of manures (pH of 5 to 6) can result in drastic decreases in emission of both NH_3_ and greenhouse gases [18, 19]. Covering stores of animal waste with a physical barrier can also drastically reduce NH_3_ emissions [20, 21]. This is a legal requirement in the UK for pig slurry stores, but beyond that, implementation is limited due to infrastructure costs. While these improvements can significantly reduce waste from stored animal manures, there are still large emissions of NH_3_ associated with animal barns, such as poultry farms. Poultry housing and manure storage account for 10.4 kt N of NH_3_ emissions annually in the UK, largely as a result of ventilation of barns [9].

Research has been conducted on NH_3_ recovery technology using acid stripping and gas permeable membrane technology to scrub NH_3_ from ventilated air from barns. NH_3_ recovery rates of 88–100% have been reported for poultry manure and pig slurry [22, 23]. Here, NH_3_ pollution stripped from the air can become a useful product, in the form of an ammoniacal salt or aqueous solution. Even in its most simple design, passing NH_3_-rich air through a column of water is enough to strip out almost 100% of the NH_3_, converting it to NH_4_^+^ [24]. The main limitations of these systems are the cost of installation, and logistical challenges in applying the captured NH_3_ in an economically beneficial way.

This study aims to assess the feasibility of a low-cost and low-tech method of NH_3_ emission capture from an agricultural point source (chicken manure) using a water column bubbling technique. The secondary aims of the study are to assess (i) the suitability of several scrubbing solutions for NH_3_ capture, and (ii) the suitability of these solutions for application to agricultural fields or treatment ponds as a replacement (or supplemental) N fertiliser.

## Method

### Ammonia capture

Chicken manure (80 kg fresh weight) was collected by removal conveyor belts from deposits directly under caged hens at Glenrath Farms (West Linton) in the Scottish Borders (no permits were required for this work as manure from a poultry unit is not regulated by the Environmental Permit for the poultry site, Environment Agency). The collected sample contained a mixture of chicken faeces, chicken feed and chicken bedding (straw and sawdust). This was stored in sealed plastic bags and transported to greenhouse facilities at the UK Centre for Ecology & Hydrology (Edinburgh). Sixty kg of chicken manure was added to a lid-sealed 90 L PVC plastic container. Four holes were drilled into the lid of the bin and 1000 mm length of 8 mm ID PVC hosing was attached to each hole via plastic grommet connectors. Each tube was then connected to an air pump (Model type TD-3LS, Brailsford & Co, Antrim, USA), which was then fitted with a further 1000 mm of 8 mm ID plastic hosing that directed the pumped air into four separate solution containers. The flow rates of the air pumps were checked for equivalence and were set to run with a flow rate of 0.8 L min^-1^.

Five high-density polyethylene 5 L plastic jerry cans were filled with 5 L of separate solutions (Table 1), namely i) 0.121 M HNO_3_, ii) 0.061 M Ca(NO_3_)_2_, iii) a mix solution of 0.491 M Ca(NO_3_)_2_ and 0.024 M HNO_3_ and two containing only deionised H_2_O. The three nitrate solutions were chosen so that end products would replicate commonly used fertilisers such as ammonium nitrate and calcium ammonium nitrate, while ammonium solution in water (ammonium hydroxide) would be similar in nature to hydrolysed urea (after application of urea to wet soils). Tubing from the output of the air pumps was placed into four of the jerry cans, with the exception of the control container (one of those containing only deionised H_2_O), which was left uncapped and open to the atmosphere for the duration of the experiment to act as a background. The hosing was suspended 1 cm from the bottom of the containers, and the caps were loosely sealed with Parafilm^®^ and tape to prevent contamination from external air.

**Table 1:** A summary of the solutions used to strip ammonia (NH_3_) from air flows from the chicken manure. Solutions were stored in 5 L plastic jerry cans for the duration of the capture period. Abbreviations for the NH_4_^+^ containing solutions are given to refer to the end-product after NH_3_ stripping occurred.

Compound Name	Formula	Molar Conc. (M)	End Product
Nitric Acid (aq)	HNO_3_	0.121	N_AN
Calcium Nitrate (aq)	Ca(NO_3_)_2_	0.061	N_CAN
Nitric Acid and Calcium Nitrate (aq)	HNO_3_ & Ca(NO_3_)_2_	0.024, 0.491	N_CAN_acid
De-Ionised water	H_2_O	NA	N_DI
Control (De-Ionised water)	H_2_O	NA	Control

During a period of 56 days from 10/10/21 to 15/12/21, 20 mL samples of the nutrient solution were taken for analysis every week for 4 weeks, then one month after for the final sample. The samples of nutrient solution were frozen immediately after collection and stored at -18 °C until further processing up to three months later. Concentrations of NH_4_^+^ and NO_3_^-^ in the solutions were measured using a SEAL AQ2 discrete analyser (SEAL Analytical, US) fitted with a cadmium coil. The widely used phenol-hypochlorite (for NH_4_^+^) and sulfanilamide (NO_2_^-^ & NO_3_^-^ after cadmium coil reduction) methods [25] were used to provide the relevant colourimetry reactions. The pH of these nutrient solution samples was taken using a pH meter (MP220, Mettler Toledo, Columbus, Ohio, USA).

### Practical application of fertiliser nutrient solution

The nutrient solutions collected during the ammonia capture stage of the experiment were applied to three plant types to test the feasibility of using the collected nutrient solutions as a N fertiliser. The plants used were the common grass (*Lolium Perenne*) which is widely used for livestock grazing, and two aquatic plants (*Taxiphyllum Barbieri* and *Salvinia auriculata*) typically grown in aquariums to help improve N removal. These plants were grown under artificial LED light (white, mimicking solar radiation), receiving 12 h of light over a 24-h period. All nitrogen applications followed a similar timeframe and N additions, described in Tables 2 and 3. For each plant, fifteen pots were divided into five sets of three (5 treatments, 3 replicates). Each set of replicates was fertilised with only one of the nutrient solutions provided from the initial phase of the experiment. This allowed for three replicates for each of the four nutrient solutions and a control set, which was only given DI H_2_O. Quantities of N solution applied were calculated from the final concentrations of both NH_4_^+^ and NO_3_^-^ present in solution to ensure total N availability was equal among treatments.

**Table 2:** Fertilisation and harvest schedule for grass growth experiment.

Date	Activity	Total Nutrient Solution applied (kg N ha^-1^)	Total N applied from Nutrient Solution (g)
01/12/21	Seed propagation		
01/02/22	Initial Cutting + Fertilised	50	0.043
17/02/22	Grass Harvest 1 + Fertilised	75	0.065
02/03/22	Grass Harvest 2 + Fertilised	75	0.065
10/03/22	Fertilised	75	0.065
21/03/22	Grass Harvest 3		

**Table 3:** Date and action performed on aquatic plants including nutrient solution application during the growth experiment.

Date	Activity	Total Fertiliser content of nutrient solution applied (g N L^-1^)	Total N applied from Nutrient Solution (g N)
01/12/21	Plants weighed and propagated into pots		
01/02/22	Fertilised	0.1	0.043
17/02/22	Fertilised	0.2	0.087
02/03/22	Fertilised	0.3	0.130
10/03/22	Fertilised	0.4	0.173
21/03/22	Plants Weighed & dried		
25/03/22	N content analysed		

### Grass planting

Grass seed (3.2 g) was planted into pots of 10.5 cm diameter and 12 cm depth with 300 g of topsoil compost from a well-mixed bag, bought from a local garden centre. Each set of three replicate pots (15 total) was placed in individual trays to ensure that any water drainage did not mix with or contaminate neighbouring pots. The grass was cut with hand scissors to 2 cm shoot length for each harvest. Pots were watered with 40 mL of DI H_2_O twice a week to account for water uptake and evaporation. The fertilisation and harvest cycle for the grasses is outlined in Table 2. The grass from each harvest was dried at 65 °C for 48 h and weighed. The nitrogen % of harvests 1, 2 and 3 were analysed using a Thermo Fisher Scientific Flash SMART with a stainless-steel column and prepacked quartz reaction tube.

### Aquatic plant preparation

10.0 g (fresh weight) of *Salvinia auriculata* was placed in each 250 mL volumetric flask with 15 g aquarium soil and 200 mL of DI H_2_O. A fresh weight of 1.0 g of *Taxiphyllum Barbieri* was placed in each 200 mL plastic beaker along with 15.0 g of aquarium soil and 100 mL of DI H_2_O. To obtain the final weights of the water plants they were carefully dried with paper towels to remove as much surface water as possible, without damaging the plants, then air dried for 24 h.

Aquarium soil contained N (0.332%), P (0.094%), K (1.09%), Mg (1.9%), Fe (1.52%), Mn (0.03%). An additional 1 mL of plant nutritional solution was added to the pots which contained a diluted solution (0.5 mL in one litre of DI water) of K (0.8%), Mg (0.4%), S (0.9%), B (0.004%), Cu (0.006%), Fe (0.07%), Mn (0.04%), Mo (0.002%), Zn (0.002%). These additions ensured that the aquatic plants had all nutrients required to grow in the conditions provided for the duration of the experiment.

### Statistical analysis

Data was analysed using the statistical software R, version 4.1.0 [26]. Standard deviation is used to illustrate uncertainty around the mean of data, assuming a gaussian distribution. Two-tailed t-test significance values (p) are provided for comparisons of treatments, using the “*t*.*test”* function from the R package “*stats”* [26].

## Results

### Ammonia capture

**Fig 1:**
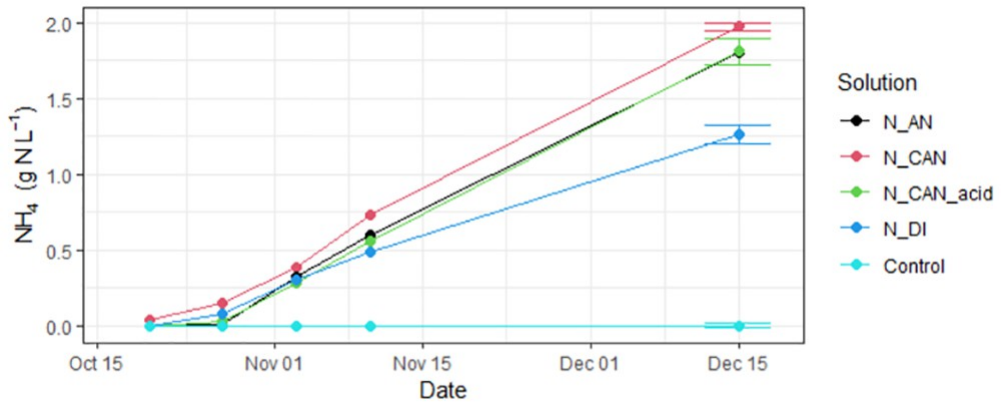
Concentrations of ammonium (NH_4_^+^) present in the stripping solutions (with nitric acid (N_AN), NH_4_^+^ with calcium nitrate (N_CAN), NH_4_^+^ with calcium nitrate and nitric acid (N_CAN_acid), NH_4_^+^ in DI water (N_DI) and the DI water that had acted as a control in the experiment (control)) during the duration of the NH_3_ stripping phase. Error bars represent the standard deviation of three replicates (data presented in Table S1 in S1 File).

After the NH_3_ capture phase, 5 solutions had been collected in which the NH_3_ had converted to NH_4_^+^. These were NH_4_^+^ with nitric acid (N_AN), NH_4_^+^ with calcium nitrate (N_CAN), NH_4_^+^ with calcium nitrate and nitric acid (N_CAN_acid), NH_4_^+^ in DI water (N_DI) and the DI water that had acted as a control in the experiment (Control). During the capture phase, NH_4_^+^ concentrations increased steadily in all of the solutions into which the air from the chicken manure storage had been pumped (Fig 1). Concentrations of NH_4_^+^ in the Control solution were below detection limits of the analysis method (0.02 mg L^-1^), thus we conclude that any contamination was negligible in the experiment. During the experiment, approximately 64 m^3^ of air was pumped through each solution resulting in a total capture of 9.05, 9.87, 9.05 and 6.33 g of NH_4_^+^ in the N_AN, N_CAN, N_CAN_acid and N_DI solutions after 56 days, respectively (Table 4). Of all the solutions, the CAN mixture had the highest NH_3_ capture (1.97 g L^-1^), followed closely by the AN and CAN_acid solutions which were almost identical in magnitude (1.81 g L^-1^). The N_DI solution captured the least NH_3_, with a final concentration of 1.27 g L^-1^, approximately 64% of that recovered in the CAN solution.

**Table 4:** Nitrogen content of the stripping solutions after 56 days of NH_3_ stripping.

	pH	NH_4_^+^ (g-N l^-1^)	NO_3_^-^ (g-N l^-1^)	Total N (g-N l^-1^)	Total NH_4_-N (g-N)	Total N (g-N)
AN	8.00	1.81	1.47	3.28	9.05	16.40
CAN	7.29	1.97	2.16	4.13	9.85	20.70
CAN_acid	7.40	1.81	2.05	3.86	9.05	19.30
N_DI	8.86	1.27	0	1.27	6.35	6.40
Control	6.80	0	0	0	0	0

**Fig 2:**
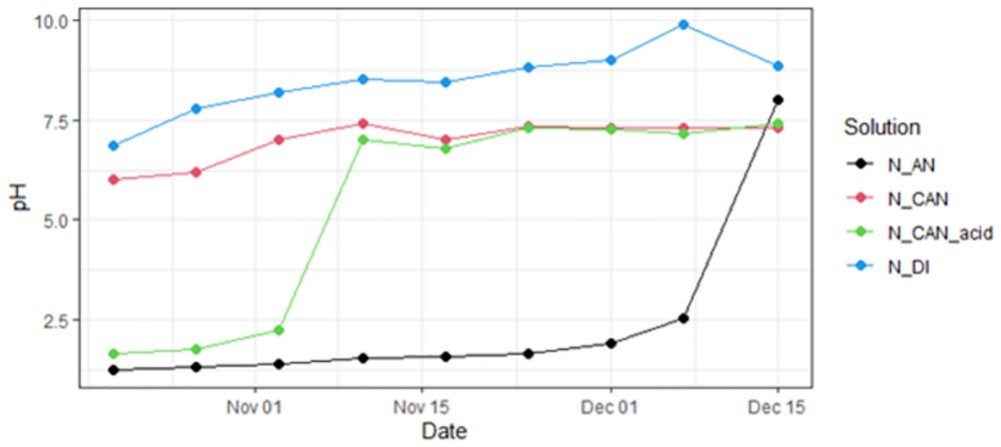
The pH in the stripping solutions during the duration of the NH_3_ stripping phase (data presented in Table S2 in S1 File).

At the beginning of the experiment, the solutions had very different pH values (Fig 2). During the NH_3_ collection, pH changes were observed in each of the solutions. The nitric acid used to make AN had a very low pH (1.25), as did the acidified calcium nitrate solution (1.65). The calcium nitrate solution was slightly acidic (6.01) while the DI water was close to neutral (6.8). During the course of the experiment, each of the solutions behaved differently as NH_4_^+^ concentrations increased (Fig 2). While the pH of N_DI and CAN solutions increased over time, this was a relatively slow change. Once the CAN_acid solution reached a 0.3 g N L^-1^ concentration for NH_4_, there was a sharp increase in pH. This same increase occurred in the AN solution, though only after the NH_4_^+^ concentration crossed the 1 g N L^-1^ threshold. All solutions exposed to the NH_3_ source reached an alkaline pH of between 7.4 to 8.9 by the end of the experiment (Fig 2, Table 4).

### Practical application of fertiliser nutrient solution

**Fig 3:**
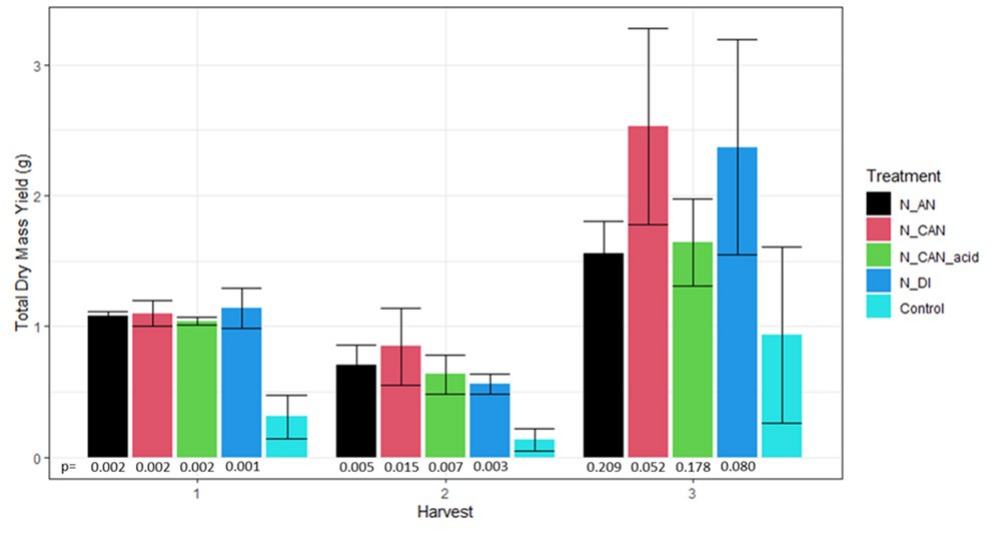
Dry mass yields of three harvests of *Lolium Perenne* grass after treatment with fertiliser solutions generated after NH_3_ stripping (as detailed in Table 2). Error bars represent the standard deviation of three replicates. Two-tailed t-test significance values (*p*) are provided of comparisons of treatment replicates with control replicates for each harvest (bottom) (Data presented in Table S3 in S1 File).

The grass seed was initially allowed to germinate and grow over a 62-day period before the start of the experiment. An initial harvest showed that the pots were all relatively similar in yields before fertiliser application (harvest mean of 13.4 g, std dev = 2.0 g). For the three harvests after N application, yields were larger for all treatments than those measured in the control plots, which were stunted in growth in comparison (Fig 3). In the first harvest, mean dry yields of grass ranged from 1.04 to 1.14 g. During the second harvest there was slightly more difference in the yields between treatments, though these differences were not larger than the deviation between replicates with yields ranging from 0.56 to 0.84 g. During the third harvest, yields from the N_CAN and N_DI treated crops (2.5 and 2.4 g of dry mass, respectively) were considerably higher than the N_AN and CAN_acid treated plots (1.5 and 1.6 g of dry mass, respectively). Overall, the CAN treatment resulted in the highest total dry yield at 1.49 g, followed by the N_DI (1.36 g), N_AN (1.1 g) and CAN_acid (1.1 g) treatments. The mean yield for the control plots was 0.46 g and positive yield response for all treatments was statistically significant (*p* < 0.1) with the exception of N_AN and N_CAN_acid in the third harvest, largely due to the large variability observed in the control plots (Fig 3).

**Fig 4:**
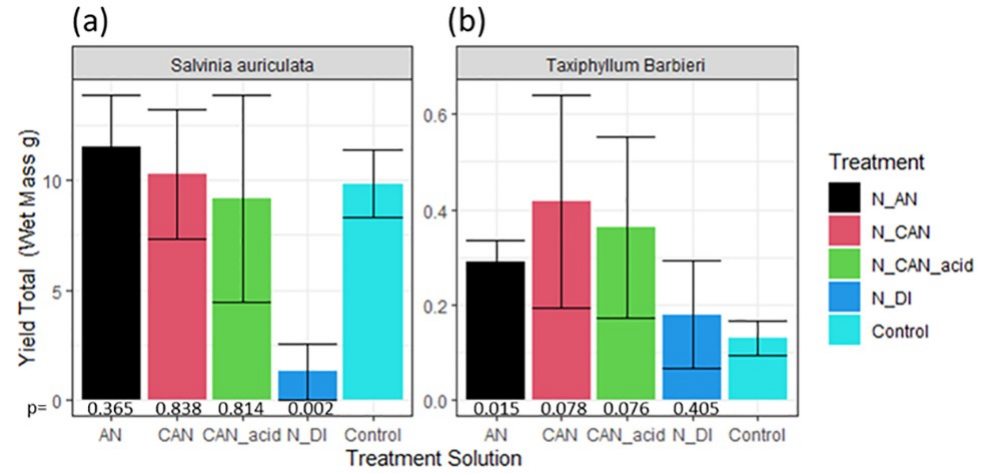
The change in fresh mass of (a) *Salvinia auriculata* and (b) *Taxiphyllum Barbieri* over 115 days, after treatment with fertiliser solutions generated after NH_3_ stripping (as detailed in Table 3). Error bars represent the standard deviation of three replicates. Two-tailed t-test significance values (*p*) are provided of comparisons of treatment replicates with control replicates for each harvest (bottom). (Data presented in Table S4 in S1 File).

For the aquatic plants, total change in fresh mass of the *Salvinia auriculata* over the 115 day period ranged from 1.29 to 11.5 g. The mean growth in the control plots (9.8 g) was relatively close in magnitude to the AN (11.5 g), CAN (10.28 g) and CAN_acid (9.14 g) treated replicates which did not vary significantly from the control plots (Fig 4a); however, the N_DI treatment resulted in a statistically significant negative impact on plant growth over the period (*p* = 0.002), in which the net change in the fresh weight of the *Salvinia auriculata* was 1.29 g, approximately 13% of the growth observed in the control. It was also noted that the leaves of the *Salvinia auriculata* exposed to the N_DI treatment had browned, and some had begun to rot, suggesting the treatment was seriously harming the plant.

Total change in fresh mass of the *Taxiphyllum Barbieri* over the 115-day period ranged from 0.18 to 0.42 g for the fertiliser treatments, while it remained low at 0.13 g for the control replicates (Fig 4b). All treatments had a positive effect on growth of the *Taxiphyllum Barbieri*, though due to high variability, only the N_AN treatment showed a significant effect (*p* = 0.015). The addition of N_DI had the smallest impact of the fertiliser solutions, with a negligible impact on yield in comparison to the control (*p* = 0.405). There was no visible difference in the health of the plants over this timeframe.

**Fig 5:**
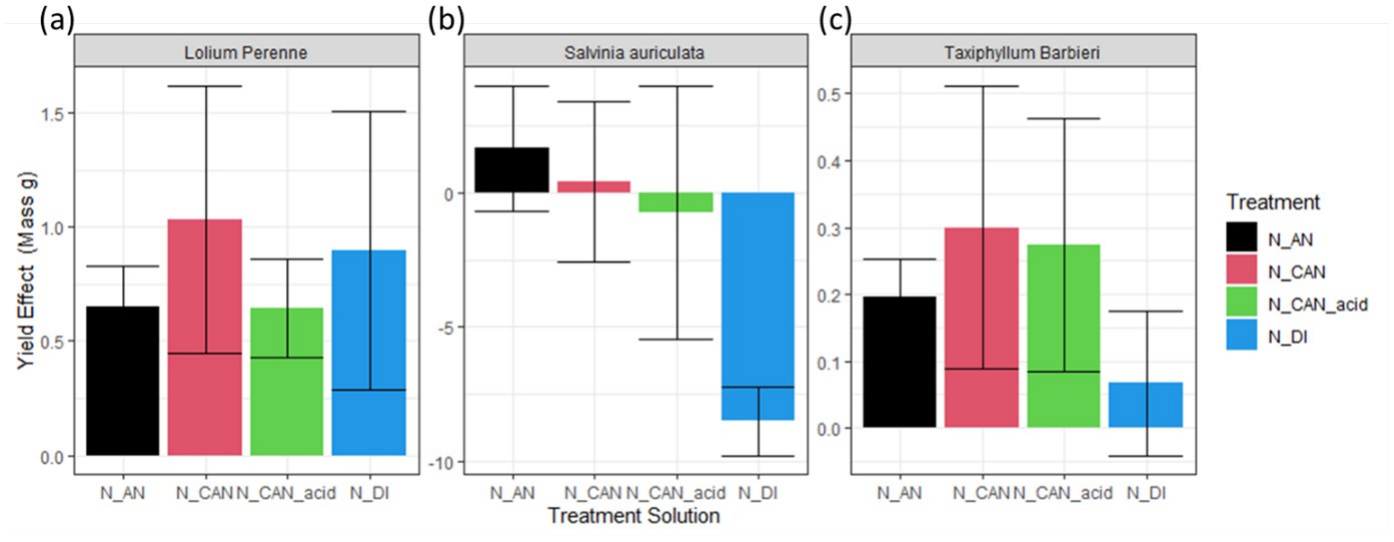
The treatment effect of each fertiliser addition to the three crop types in this experiment. Treatment effect is calculated by subtracting the mass harvested from control replicates from treatment replicates. Error bars represent the standard deviation of three replicates.

In terms of treatment effect, there was a mixed response to the different fertiliser solutions. When adding the solutions with stripped NH_4_^+^ to the potted grass, all were successful at increasing yield in comparison to control replicates, more than doubling harvests for all solutions (Fig 5a). The form of N in these solutions does not seem to have affected the impact on crop growth. However, for the aquatic plants there is a clear difference between the nitrate containing solutions (N_AN, N_CAN and N_CAN_acid) in comparison to the NH_4_^+^ only solution (N_DI). The application of N_DI had a strong negative effect on the Salvinia auriculata (Fig 5b), while it had negligible impact on *Taxiphyllum Barbieri growth*, in contrast to the other fertiliser solutions (Fig 5c).

## Discussion

### The capture and storage of NH_3_

The results of this feasibility study have provided evidence of several aspects of NH_3_ capture and utilisation that may be useful when developing NH_3_ capture technologies in the future, especially when considering further utilisation in agriculture. While the absolute overall capture rate of NH_3_ (and conversion to NH_4_^+^) is not quantified in this study, we know from previous studies that, simply using water, it is possible to strip almost 100% of NH_3_ from air [24] and capture rates depend primarily on the design of the capture mechanism. We also know from previous studies that NH_3_ gas is highly soluble, and that a maximum of approximately 320 g of NH_3_ could be dissolved in one litre of water at 25 °C. In comparison, 2130 g of ammonium nitrate (NH_4_NO_3_) can also be dissolved in a litre of water [27]. This gives a maximum N content of 263.5 g L^-1^ and 745.7 g L^-1^ for NH_3_ and AN, respectively. This high solubility in water is the reason that simple, low-tech solutions for NH_3_ capture like air bubbling or mist scrubbing [28] are highly effective (>80%) at removing NH_3_ from the atmosphere at low cost.

In this study, 64.5 m^3^ of air passed through water resulted in the capture of 6.33 g of NH_3_-N over a period of 56 days (capture of 0.11 g day^-1^). Hypothetically, using simplistic assumptions, if this absorption rate remained stable, it would take over 6.5 years to saturate 5 litres of water with NH_3_, showing that in order to be successful, a significant airflow of elevated NH_3_ concentration is required to produce meaningful quantities of NH_4_ in solution. The highest absorption rate of NH_3_ in this experiment was of the CAN solution, capturing approximately 0.15 g of NH_3_-N per m^-3^ of air bubbled through the solution. Assuming a 100% capture rate, we can estimate that the mean air concentration of NH_3_ passing through the system was at least 201 ppm which is in line with what would be expected from a manure bin. It has been reported in literature that NH_3_ concentrations in chicken barns in the UK can reach 20–52 ppm in the winter when the barns are shut to the elements, and 12–25 ppm in the summer [29]. Higher concentrations are not uncommon [30]. At these high NH_3_ concentrations and with higher air flow rates, the capture rate N from simple stripping systems would be significantly higher as demonstrated in a review by Pandey and Chen (2021) [31], where NH_4_-N recovery exceeds 90% in the majority of examples.

We confirm in this study that solutions acidified with nitrate (NO_3_^-^) are able to better strip NH_3_ from the atmosphere than pure water. This comes as no surprise, as acidified solutions are often used to strip NH_3_, most commonly in the form of sulphuric acid [31]. The value of using nitric acid instead of sulphuric acid, is that a more concentrated content of N can be produced in the solution, thus increasing the value of the captured NH_3_ as an N fertiliser product. The addition of NO_3_^-^ more than doubles the maximum solubility of total N in the form of dissolved NH_4_NO_3_, and our study suggests that the addition of NO_3_^-^ strongly increases the capture rate of NH_3_ in the stripping process as each of the AN, CAN and CAN_acid solutions performed considerably better than the N_DI. In theory, a highly concentrated nitric acid solution could capture a large quantity of NH_3_ (373 g L^-1^), though the use of large quantities of such concentrated acids would pose serious risk of harm to users. The alternative to using acid, is to use a dissolvable nitrate powder such as calcium nitrate Ca(NO_3_)_2_. The pH of dissolved calcium nitrate is much safer to handle (pH = 5.5) than that of strong acids, and high concentrations of nitrate in solution can also be achieved (saturation of 1440 g of Ca(NO_3_)_2_ per litre of water [27]). In this study the dissolved calcium nitrate had the highest NH_3_ capture rate of all the solutions, though this came with a large drawback, the precipitation of calcium (or calcium salt). As the CAN and CAN_acid solutions became more alkaline in nature, the calcium began to precipitate out of the solution around the air inlet of the tubing. This precipitation clogged the tubing and needed to be manually removed. In a larger barn-scale system this would be a serious drawback of using calcium salt solutions (or solutions of other elements expected to precipitate) as it could result in expensive mechanical failures and clogging of pipes.

The capture and storage of high concentration N solutions does present some risk for farmers. Where only water is used to capture NH_3_, if saturation was reached, gaseous NH_3_ would be released from the system as uptake and emission reaches equilibrium. However, where nitrate (or similar anions) are present, ammoniacal salts can precipitate from the solution once threshold pH or temperature conditions are met. Ammoniacal salts, especially of nitrate, pose an explosive risk. The precipitation and collection of ammonium nitrate also provides the possibility of the generation of undocumented AN fertiliser, which is a highly regulated substance due to its explosive properties. To combat AN being used as an explosive, calcium can be added to form CAN, thus reducing its explosive properties; however, CAN can still be used in the manufacturing of illegal explosives.

The use of ponds to filter out nitrogen pollution via natural processes is becoming more common as it is seen as a cost-effective way to deal with nitrogen and phosphorus overspill from farming [32]. Here, nitrogen and phosphorus hungry aquatic plants can consume N and P and prevent loss to the environment. In theory, a low cost and low-tech method of dealing with NH_3_ from point sources such as chicken barns would be to strip it out using a water solution, and feed it into a pond with nitrogen hungry aquatic plants, which could then be harvested for use (e.g. animal feed). Storage of the NH_4_^+^ is then no longer a safety concern in terms of explosion risk. In this study we observed the effect of exposing aquatic plants to captured NH_4_^+^ solution. The reaction of the *Salvinia auriculata* to the exposure of the N_DI solution is typical of aquatic plants. In aquatic environments, plants are much more sensitive to ammonia toxicity compared to soil grown plants. The *Taxiphyllum Barbieri* was more resilient to the NH_4_^+^ addition in comparison, but did not benefit substantially from it in terms of growth. The sensitivity of aquatic plants to ammonium concentrations with the added risk of algal blooms in the storage ponds [33] may make this activity difficult to logistically balance and maintain.

### The utilisation of captured NH_3_

In current scrubbing systems, it is typical for NH_3_ to be captured from chicken barns using a sulphuric acid mist, then the captured solution is flushed into sewage. While this practice is semi-beneficial to the environment, it comes at a cost to the farmer, and to some extent the taxpayer, in the form of sewage treatment requirements. Captured NH_3_ (or NH_4_^+^) is often limited in usefulness for several reasons. For maximum efficiency in scrubbing, solutions that contain less NH_4_^+^ will capture the NH_3_ from the atmosphere more effectively. Thus, when using water or acid scrubbing methods, it’s uncommon to collect high concentrations of N in solution. Concentrating NH_4_^+^ in dilute systems (after collection) is practically difficult and economically restrictive. Using temperature controls (e.g. cooling) to precipitate ammoniacal salts at scale would be impractical and cost-restrictive to the vast majority of farmers with NH_3_ point sources. This makes long-term storage or transportation of the low N solution difficult and potentially expensive to manage.

Ideally, the solution of captured NH_3_ would be immediately useful at a local level to the farmer. For example, applying a low N solution regularly to fields near a point source of NH_3_ emissions via irrigation or sprinkler systems is one beneficial scenario, as the farmer saves on fertiliser costs as well as reducing environmental footprint. This scenario is best fitting for crops which already require a lot of irrigation and will benefit from the regular application of extra water. A prime example of this would be in the application of captured N to fodder crops in a dry climate. However, applying regular applications of NH_4_^+^ solutions to soils comes with the possibility of further NH_3_ emissions, this is especially true in warm dry soils where a fraction of the NH_4_^+^ will re-volatilise into NH_3_ and re-emit from the soil, similar to application of urea or animal urine. While we have shown in this study that the NH_4_^+^ enriched water can match the other solutions that are less prone to NH_3_ volatilisation (AN and CAN) for nitrogen use efficiency, this efficiency would be highly dependent on weather.

Other crop scenarios which require regular N input include horticulture, glasshouse agriculture and other controlled environment agriculture systems like vertical farming or industrial algae production. The limiting factor here is similar to that of the sensitivities to aquatic plants. High NH_4_^+^ concentrations in solution will damage sensitive plants in hydroponic systems, or waterborne algae. In the hydroponic industry, it is typical for nitrate concentrations to exceed those of NH_4_^+^ in fertilisers by a factor of 10:1 to avoid ammonium toxicity. However, this study demonstrates that potted plants in soil, such as those grown in horticultural glasshouse conditions, respond well to NH_4_^+^ in solution, as well as those compounds where NH_4_^+^ was balanced with NO_3_^-^. Horticulture growing methods commonly include drip trays and ebb and flow irrigation, in which crops are exposed to dilute forms of N and other nutrients. These conditions would be ideal for the utilisation of NH_3_ solutions if growing location was local to the point source location where emissions were captured.

### The economics of captured NH_3_

There are several factors to consider when establishing the monetary value of NH_3_ capture and utilisation in agriculture. The primary concern, and the main driving force behind if action is taken, is whether such activities were financially beneficial to farmers. In the UK, it’s estimated that 58.5 kt NH_3_ from animal housing/barns and 19.8 kt NH_3_ from manure storage is lost to the environment annually [9]. These sources are considered the easiest to apply methods to capture NH_3_ as airflows and ventilation can be managed. Based on current prices of mineral N fertiliser (AHDB, May 2023), one kg of N costs approximately £1.48 This NH_3_ accounts for 64.5 kt N, the equivalent of £95.5 million of mineral fertiliser. This is the equivalent of 9% of all mineral fertiliser applied in the UK (2019, BSFP [34]), or 46% of all urea-based mineral fertilisers applied in the UK.

While there is a large degree of uncertainty in estimating the financial benefits of NH_3_ capture systems at any given farm due to the wide variety of factors that need to be considered, we can make some very simplistic calculations using values from literature to assess an approximate magnitude of costs. Using the daily NH_3_ production rates of pigs reported in Cao et al. (2020) [19], an approximation of 0.6 kg to 2 kg of NH_3_ is emitted per pig every year. For a pig farm with 1000 pigs, this would mean the equivalent of 500 to 1600 kg N lost every year, amounting to £740 to £2370 of an equivalent mass of N in the form of mineral fertiliser (at £1.48 per kg N). The Environment Agency estimates an NH_3_ emission factor of 0.034 kg NH_3_ per broiler chicken per year in the UK. Assuming 20 to 50 thousand birds for a large-scale chicken farm, this amounts to 560 to 1400 kg N per farm per year, or approximately £830 to £2070 worth of mineral N fertiliser (at £1.48 per kg N). While not insignificant, the value of this lost N is unlikely to be recuperated once the electricity costs of ventilation are accounted for. Industrial air scrubbers can consume upwards of 100 kWh d^-1^, the equivalent of over £12,000 a year at current (October 2023) electricity prices in the UK (£0.34/kWh, Energy Saving Trust). These prices could be mitigated if renewable energy is produced by the farmers on-site (e.g. solar and wind); however, these methods are not without costs of their own.

In terms of overall value of NH_3_ removal, the societal costs should also be considered. With N pollution, it is hard to put an exact cost on emissions due to the nitrogen cascade, in which nitrogen will form many different compounds before eventually becoming inert dinitrogen (N_2_) gas again (e.g. NH_3_ to NO_3_^-^ to N_2_O). Also, the true cost of the impacts depend highly on where pollution is released and how humans and the environment are exposed in these areas. In Van Grinsven et al. (2013) [16], the cost of NH_3_ released from sources across the EU was valued at 2 to 20 € per kg N emitted in terms of impact on human heath (particulate matter exposure). The impact on ecosystems was estimated to be 2 to 10 € per kg N. In the above pig and chicken farm examples (500 to 1600 kg N lost per year), this would result in societal costs ranging from less than £2000 to over £60,000 in today’s money (Apr 2023, € to £ = 0.87 conversion). The cost of the impacts of NH_3_ emissions depend greatly on farm location and wind dynamics. For example, a high NH_3_ emitting farm upwind of a densely populated urban area, or a nitrogen sensitive environment, would have a far greater societal impact and perceived cost than that of a similar farm where winds predominantly carried emissions out to arable land where NH_3_ deposition can act as additional fertiliser.

## Conclusion

In this study we show that NH_3_ can be captured from manure, and that solutions of common fertiliser compounds such as ammonium nitrate and calcium ammonium nitrate can be replicated by binding captured NH_3_ to solutions of nitrate. Our results suggest that dissolved calcium nitrate is just as effective at scrubbing NH_3_ from the atmosphere as nitric acid but could do so at a near neutral pH. In terms of use in agriculture, applying all of the fertiliser solutions produced by NH_3_ capture in this study was successful in increasing the yield of grass grown in soil, including the ammonium only solution (N_DI). Here we demonstrate that captured NH_3_ can be used viably as a fertiliser in the field if the mechanisms to deliver it are present. We have shown that aquatic plants can be more sensitive to NH_4_^+^ in solution, and that additional buffers (such as the presence of nitrate) are required for aquatic plants to survive NH_4_^+^ fertilisation. In conclusion, we highlight that the capture and utilisation of NH_3_ emissions from point sources is possible using very basic apparatus; however, the overall economic practicality of this method will depend heavily on any given scenario. Regardless of these difficulties, a low-cost and low-tech method to achieve this may be viable, and so long as innovators understand the difficulties like those presented in this study, future trials may provide low-tech/low-cost methods by which to achieve environmental savings and value for farmers.

## Supporting information

S1 File A low tech, low cost method to capture point-source ammonia emissions and their potential use as a nitrogen fertiliser. (DOCX)
