# Supplementary material for: A low-tech, low-cost method to capture point-source ammonia emissions and their potential use as a nitrogen fertiliser
Source: PLoS One. 2024 Jan 31;19(1):e0296679. doi: 10.1371/journal.pone.0296679 (PMC10830035; doi:10.1371/journal.pone.0296679)
Supplement: S1 File — (DOCX) [file pone.0296679.s001.docx]

**A low tech, low cost method to capture point-source ammonia emissions and their potential use as a nitrogen fertiliser**

**Supplementary Materials**

**Table S1** Concentrations of ammonium (NH_4_^+^) and nitrate (NO_3_^-^) measured from the ammonia capture solutions at the end of the 56-day capture period, presented in Fig 1.

| Replicate | Solution | Test | Sample_Conc | Sample_Unit |
| --- | --- | --- | --- | --- |
| 1 | N_CAN | Ammonium | 1.98 | g N/L |
| 2 | N_CAN | Ammonium | 2.00 | g N/L |
| 3 | N_CAN | Ammonium | 1.94 | g N/L |
| 1 | N_AN | Ammonium | 1.91 | g N/L |
| 2 | N_AN | Ammonium | 1.73 | g N/L |
| 3 | N_AN | Ammonium | 1.78 | g N/L |
| 1 | N_CAN_acid | Ammonium | 1.85 | g N/L |
| 2 | N_CAN_acid | Ammonium | 1.72 | g N/L |
| 3 | N_CAN_acid | Ammonium | 1.87 | g N/L |
| 1 | N_DI | Ammonium | 1.32 | g N/L |
| 2 | N_DI | Ammonium | 1.27 | g N/L |
| 3 | N_DI | Ammonium | 1.20 | g N/L |
| 1 | Control | Ammonium | -0.01 | g N/L |
| 2 | Control | Ammonium | 0.02 | g N/L |
| 3 | Control | Ammonium | -0.01 | g N/L |
| 1 | N_CAN | Nitrate | 2.16 | g N/L |
| 2 | N_CAN | Nitrate | 2.19 | g N/L |
| 3 | N_CAN | Nitrate | 2.14 | g N/L |
| 1 | N_AN | Nitrate | 1.50 | g N/L |
| 2 | N_AN | Nitrate | 1.49 | g N/L |
| 3 | N_AN | Nitrate | 1.43 | g N/L |
| 1 | N_CAN_acid | Nitrate | 2.10 | g N/L |
| 2 | N_CAN_acid | Nitrate | 2.01 | g N/L |
| 3 | N_CAN_acid | Nitrate | 2.03 | g N/L |
| 1 | N_DI | Nitrate | 0.00 | g N/L |
| 2 | N_DI | Nitrate | 0.00 | g N/L |
| 3 | N_DI | Nitrate | 0.00 | g N/L |
| 1 | Control | Nitrate | 0.00 | g N/L |
| 2 | Control | Nitrate | 0.00 | g N/L |
| 3 | Control | Nitrate | 0.00 | g N/L |

**Table S2** Solution pH measured during the ammonium capture period as presented in Fig 2.

| Date | Solution | pH |
| --- | --- | --- |
| 20/10/2021 | CAN | 6.01 |
| 27/10/2021 | CAN | 6.199 |
| 03/11/2021 | CAN | 7.01 |
| 10/11/2021 | CAN | 7.41 |
| 17/11/2021 | CAN | 6.99 |
| 24/11/2021 | CAN | 7.34 |
| 01/12/2021 | CAN | 7.31 |
| 07/12/2021 | CAN | 7.29 |
| 15/12/2021 | CAN | 7.29 |
| 20/10/2021 | AN | 1.25 |
| 27/10/2021 | AN | 1.32 |
| 03/11/2021 | AN | 1.4 |
| 10/11/2021 | AN | 1.57 |
| 17/11/2021 | AN | 1.6 |
| 24/11/2021 | AN | 1.65 |
| 01/12/2021 | AN | 1.91 |
| 07/12/2021 | AN | 2.56 |
| 15/12/2021 | AN | 8 |
| 20/10/2021 | CAN_acid | 1.65 |
| 27/10/2021 | CAN_acid | 1.77 |
| 03/11/2021 | CAN_acid | 2.25 |
| 10/11/2021 | CAN_acid | 7.02 |
| 17/11/2021 | CAN_acid | 6.8 |
| 24/11/2021 | CAN_acid | 7.31 |
| 01/12/2021 | CAN_acid | 7.28 |
| 07/12/2021 | CAN_acid | 7.14 |
| 15/12/2021 | CAN_acid | 7.4 |
| 20/10/2021 | N_DI | 6.84 |
| 27/10/2021 | N_DI | 7.78 |
| 03/11/2021 | N_DI | 8.2 |
| 10/11/2021 | N_DI | 8.52 |
| 17/11/2021 | N_DI | 8.43 |
| 24/11/2021 | N_DI | 8.81 |
| 01/12/2021 | N_DI | 9 |
| 07/12/2021 | N_DI | 9.86 |
| 15/12/2021 | N_DI | 8.86 |

**Table S3** Yields of three harvests of Lolium Perenne grass after treatment with fertiliser solutions (as presented in Fig 3). Fresh and dried mass of harvested yields are provided.

| Date | Harvest | Rep | Solution | Fresh Mass  (g) | Dried Mass  (g) |
| --- | --- | --- | --- | --- | --- |
| 17/02/2022 | 1 | 1 | CAN | 7.61 | 1.14 |
| 17/02/2022 | 1 | 2 | CAN | 8.06 | 1.17 |
| 17/02/2022 | 1 | 3 | CAN | 6.67 | 0.99 |
| 17/02/2022 | 1 | 1 | AN | 7.28 | 1.07 |
| 17/02/2022 | 1 | 2 | AN | 7.35 | 1.12 |
| 17/02/2022 | 1 | 3 | AN | 6.97 | 1.04 |
| 17/02/2022 | 1 | 1 | CAN_acid | 6.65 | 1.07 |
| 17/02/2022 | 1 | 2 | CAN_acid | 7.34 | 1.01 |
| 17/02/2022 | 1 | 3 | CAN_acid | 7.60 | 1.04 |
| 17/02/2022 | 1 | 1 | N_DI | 7.03 | 1.02 |
| 17/02/2022 | 1 | 2 | N_DI | 7.77 | 1.08 |
| 17/02/2022 | 1 | 3 | N_DI | 7.56 | 1.31 |
| 17/02/2022 | 1 | 1 | Control | 2.75 | 0.16 |
| 17/02/2022 | 1 | 2 | Control | 2.18 | 0.28 |
| 17/02/2022 | 1 | 3 | Control | 2.94 | 0.49 |
| 02/03/2022 | 2 | 1 | CAN | 4.46 | 1.18 |
| 02/03/2022 | 2 | 2 | CAN | 3.87 | 0.72 |
| 02/03/2022 | 2 | 3 | CAN | 1.67 | 0.64 |
| 02/03/2022 | 2 | 1 | AN | 4.62 | 0.84 |
| 02/03/2022 | 2 | 2 | AN | 4.07 | 0.54 |
| 02/03/2022 | 2 | 3 | AN | 3.99 | 0.74 |
| 02/03/2022 | 2 | 1 | CAN_acid | 3.96 | 0.80 |
| 02/03/2022 | 2 | 2 | CAN_acid | 4.52 | 0.57 |
| 02/03/2022 | 2 | 3 | CAN_acid | 3.19 | 0.53 |
| 02/03/2022 | 2 | 1 | N_DI | 2.34 | 0.49 |
| 02/03/2022 | 2 | 2 | N_DI | 1.55 | 0.55 |
| 02/03/2022 | 2 | 3 | N_DI | 3.52 | 0.64 |
| 02/03/2022 | 2 | 1 | Control | 1.91 | 0.04 |
| 02/03/2022 | 2 | 2 | Control | 1.39 | 0.16 |
| 02/03/2022 | 2 | 3 | Control | 1.30 | 0.20 |
| 21/03/2022 | 3 | 1 | CAN | 9.29 | 2.46 |
| 21/03/2022 | 3 | 2 | CAN | 9.76 | 1.82 |
| 21/03/2022 | 3 | 3 | CAN | 8.64 | 3.31 |
| 21/03/2022 | 3 | 1 | AN | 8.97 | 1.63 |
| 21/03/2022 | 3 | 2 | AN | 9.57 | 1.27 |
| 21/03/2022 | 3 | 3 | AN | 9.49 | 1.76 |
| 21/03/2022 | 3 | 1 | CAN_acid | 9.78 | 1.98 |
| 21/03/2022 | 3 | 2 | CAN_acid | 10.37 | 1.31 |
| 21/03/2022 | 3 | 3 | CAN_acid | 9.85 | 1.64 |
| 21/03/2022 | 3 | 1 | N_DI | 9.66 | 2.02 |
| 21/03/2022 | 3 | 2 | N_DI | 9.34 | 3.31 |
| 21/03/2022 | 3 | 3 | N_DI | 9.77 | 1.78 |
| 21/03/2022 | 3 | 1 | Control | 8.88 | 0.19 |
| 21/03/2022 | 3 | 2 | Control | 9.81 | 1.13 |
| 21/03/2022 | 3 | 3 | Control | 9.66 | 1.49 |

**Table S4** Yields of three replicates of Salvinia auriculata and Taxiphyllum Barbieri after treatment with fertiliser solutions (as presented in Fig 4). Fresh mass prior to and after fertiliser addition were recorded.

| Solution | Plant Type | Rep | Start Mass  (g) | End Mass  (g) |
| --- | --- | --- | --- | --- |
| CAN | Salvinia auriculata | 1 | 9.95 | 17.06 |
| CAN | Salvinia auriculata | 2 | 10.06 | 20.89 |
| CAN | Salvinia auriculata | 3 | 10.03 | 22.93 |
| AN | Salvinia auriculata | 1 | 9.98 | 20.05 |
| AN | Salvinia auriculata | 2 | 9.97 | 20.30 |
| AN | Salvinia auriculata | 3 | 10.05 | 24.22 |
| CAN_acid | Salvinia auriculata | 1 | 10.04 | 17.05 |
| CAN_acid | Salvinia auriculata | 2 | 9.96 | 15.86 |
| CAN_acid | Salvinia auriculata | 3 | 10.01 | 24.54 |
| N_DI | Salvinia auriculata | 1 | 10.04 | 11.45 |
| N_DI | Salvinia auriculata | 2 | 10.07 | 12.60 |
| N_DI | Salvinia auriculata | 3 | 10.10 | 10.05 |
| Control | Salvinia auriculata | 1 | 10.00 | 18.22 |
| Control | Salvinia auriculata | 2 | 10.05 | 21.30 |
| Control | Salvinia auriculata | 3 | 10.09 | 20.09 |
| CAN | Taxiphyllum Barbieri | 1 | 1.01 | 1.17 |
| CAN | Taxiphyllum Barbieri | 2 | 0.98 | 1.51 |
| CAN | Taxiphyllum Barbieri | 3 | 1.00 | 1.56 |
| AN | Taxiphyllum Barbieri | 1 | 1.03 | 1.36 |
| AN | Taxiphyllum Barbieri | 2 | 1.02 | 1.32 |
| AN | Taxiphyllum Barbieri | 3 | 1.01 | 1.25 |
| CAN_acid | Taxiphyllum Barbieri | 1 | 1.02 | 1.60 |
| CAN_acid | Taxiphyllum Barbieri | 2 | 1.04 | 1.32 |
| CAN_acid | Taxiphyllum Barbieri | 3 | 1.01 | 1.24 |
| N_DI | Taxiphyllum Barbieri | 1 | 0.99 | 1.09 |
| N_DI | Taxiphyllum Barbieri | 2 | 1.02 | 1.15 |
| N_DI | Taxiphyllum Barbieri | 3 | 0.99 | 1.30 |
| Control | Taxiphyllum Barbieri | 1 | 1.01 | 1.18 |
| Control | Taxiphyllum Barbieri | 2 | 0.98 | 1.10 |
| Control | Taxiphyllum Barbieri | 3 | 0.96 | 1.06 |
